# The pro-differentiating capability of a flavonoid-rich extract of *Citrus bergamia* juice prompts autophagic death in THP-1 cells

**DOI:** 10.1038/s41598-024-70656-4

**Published:** 2024-08-28

**Authors:** Laura Musumeci, Caterina Russo, Udo Schumacher, Giovanni Enrico Lombardo, Alessandro Maugeri, Michele Navarra

**Affiliations:** 1 Department of Chemical, Biological, Pharmaceutical and Environmental Sciences, University of Messina, 98166 Messina, Italy; 2 Institute of Anatomy and Experimental Morphology, University Cancer Center Hamburg, University Medical Center Hamburg-Eppendorf, 20246 Hamburg, Germany; 3 Medical School Berlin, 10117 Berlin, Germany; 4 Department of Veterinary Sciences, University of Messina, 98168 Messina, Italy

**Keywords:** Acute myeloid leukemia, Bergamot juice extract, Flavonoids, Nutraceuticals, Differentiation, Autophagy, Biochemistry, Cancer, Oncology

## Abstract

Acute myeloid leukemia (AML) is a hematologic neoplasm, characterized by a blockage of differentiation and an unconstrained proliferation of immature myeloid cells. Recently, the survival of leukemia patients has increased thanks to the use of differentiating agents, though these may cause serious side effects. Hence, the search for safer differentiating compounds is necessary. Our aim was to assess the pro-differentiating effects of a flavonoid-rich extract of bergamot juice (BJe) in human monocytic leukemia THP-1 cells, an in vitro AML model. For the first time, we showed that treatment with BJe induced differentiation of THP-1 cells, changes in cell morphology and increased expression of differentiation-associated surface antigens CD68, CD11b and CD14. Moreover, BJe enhanced protein levels of autophagy-associated markers, such as Beclin-1 and LC3, as well as induced the phosphorylation of the MAPKs JNK, ERK and p38, hence suggesting a potential mechanism underlying its antiproliferative effects. Indeed, parallel experiments highlighted that BJe was able to hamper THP-1 cell growth. In conclusion, our study suggests that BJe induces the differentiation of THP-1 cells and reduces their proliferation, highlighting its potential in differentiation therapy of AML.

## Introduction

Acute myeloid leukemia (AML) is a malignant hematologic neoplasm characterized by the arrest of normal differentiation of immature myeloid cells (blasts), which proliferate uncontrollably, accumulating in the bone marrow and impairing the normal process of hematopoiesis. AML is one of the most common types of leukemia affecting adults^1,2^, although it can also affect children more rarely, and is associated with poor prognosis. In AML, the bone marrow produces an excessive number of blasts, substituting normal marrow tissue and hematopoietic cells, thus inducing anemia, granulocytopenia and thrombocytopenia^3^. Currently, the established protocol of AML treatment consists in a combination of the chemotherapeutic agents cytarabine and anthracyclines^4^; however, they are not devoid of severe side effects, hence compromising the overall effectiveness of these drugs.

Since a major problem in the pathophysiology of AML is the blockage of differentiation of immature progenitors, the so-called “differentiation therapy” represents a promising strategy for AML management^5^. It aims to reprogram malignant leukemic cells into functional ones, and to force them to enter the apoptotic pathway as a result of terminal differentiation. The employment of all-trans retinoic acid (ATRA) and/or arsenic trioxide in acute promyelocytic leukemia (APL) patients, brought tremendous improvements in terms of considerable remission rates and long-term survival (between 80 and 90%)^6^. Unfortunately, these compounds are only clinically successful for the APL subtype, which stands for only 5–10% of total AML cases, with no effectiveness for the other ones. Therefore, the search of new differentiation inducers for AML management, in particular for other AML subtypes, is certainly necessary.

Plant kingdom represents a treasure trove of substances that can be exploited in the prevention and/or treatment of different pathologies, including cancer^7–9^. Among these compounds, a pivotal role is occupied by flavonoids, secondary plant metabolites, very abundant in *Citrus* fruits^10^, which have been shown to possess different health-promoting properties^11,12^, including anti-tumor activities^13,14^. The pro-apoptotic, anti-proliferative and cell cycle-arresting effects of these polyphenolic compounds sustain their antileukemic potentiality^15^. In addition, several studies asserted their ability to promote differentiation in different leukemic cells^16–18^, thus suggesting flavonoids as attractive candidates in the context of the differentiation-inducing therapy for leukemia management^19^.

Among flavonoids, those retrieved from *Citrus* fruits stand out amongst others thanks to their pharmacological properties^20^. In this context, *Citrus bergamia* Risso (bergamot), a small tree belonging to the Rutaceae family, has been widely studied for the broad spectrum of biological effects showed by its derivatives^21^, with the most known being its essential oil. Bergamot juice (BJ), and more specifically the flavonoid-rich fraction of BJ (BJe) has been suggested to possess anticancer effects^22^.

In light of these considerations, we investigated whether BJe was able to induce terminal differentiation in human leukemia monocytic THP-1 cells, as in vitro model of AML, focusing on the mechanisms underlying these effects.

## Materials and methods

### *Citrus bergamia* juice extract

*Citrus bergamia* fruits, harvested in Reggio Calabria (Italy), were bought from the local markets. Upon peel removal, fruits were squeezed, and the primary juice was removed. The remaining pulp was processed with a pressing machine to retrieve the secondary juice that was extracted and concentrated. In order to retrieve the flavonoid-rich fraction, we used ethanol to perform the final elution, and the eluate was transformed into a dry powder by lyophilization. The obtained BJe powder was kept at 4° C in the dark before utilization. The extract used in the current study was the same already utilized in our previously published work^23^. Nonetheless, prior beginning this study, we carried out the quali-quantitative analysis of flavonoids present in BJe by reverse phase high performance liquid chromatograph coupled with a diode array detector (RP-HPLC-DAD) separation, confirming the same chemical composition already reported^23^. Indeed, the most abundant flavonoids of the extract were naringin and neohesperidin, accounting for about the 83% of the whole BJe, followed by neoeriocitrin. Other flavonoids identified, despite to a smaller amount, were vicenin-2, rhoifolin and neodiosmin.

### Cell cultures and treatments

The experiments were carried out using the human leukemia monocytic THP‐1 cells, originally obtained from ATCC (Rockville, MD, USA) and with an approximate doubling time of 26 h, employed as an in vitro model of AML. THP-1 cells were grown in RPMI 1640 medium supplemented with 10% (*v*/*v*) heat-inactivated fetal bovine serum (FBS), sodium pyruvate (1 mM), L-glutamine (2 mM), 2-mercaptoethanol (0.05 mM), HEPES (10 mM), penicillin (100 IU/mL) and streptomycin (100 µg/mL), glucose (2.5 g/l), at 37 °C in a 5% CO2 air atmosphere. Each reagent for cell culture was from Gibco (Life Technologies, Monza, Italy). For all experiments, THP-1 cells were treated with BJe at the concentrations that were previously described^24^.

### Nitroblue tetrazolium (NBT) reduction assay

With the aim of evaluating whether BJe was able to induce differentiation in THP-1 cells, we performed the nitroblue tetrazolium (NBT) reduction analysis, as previously explained^25^. For this assay, THP-1 cells were seeded at a density of 3 × 10^4^ cells/well (3 × 10^5^ cells/mL) in a 96-well plate and treated with 0.5, 1 and 2.5 mg/mL of BJe up to 96 h (by renewing treatment after 48 h); we used only medium for negative controls and 10 nM of phorbol 12-myristate 13-acetate (PMA) for the positive ones. After incubation, plates were centrifuged and wells washed with 100 µL of PBS; then, cells were resuspended with 100 µL of PBS containing NBT (2 mg/mL) and PMA (100 ng/mL) at 37 °C for 30 min. Subsequently, we centrifuged the plates and the newly formed diformazan crystals were dissolved with 1:3 KOH 2 M/DMSO solution. Absorbance of each well was spectrophotometrically measured by an iMark™ microplate reader (Bio-Rad Laboratories, Milan, Italy). In parallel, we also evaluated the viability of cells treated with the same concentrations and timings via the 3-(4,5-dimethylthiazole-2-yl)-2,5-diphenyl-tetrazolium bromide (MTT) test, as previously described^26^. This is because we normalized the amount of NBT reduction for the relative number of living cells, by calculating the degree of differentiation as a ratio of NBT absorbance values over MTT ones. All the experiments were performed in octuplicates repeated three times.

### Morphological analysis

In order to determine whether the extract was able to modify cell morphology, we seeded 1 × 10^6^ THP-1 cells in 100-mm dishes (3 × 10^5^ cells/mL). After overnight incubation, cells were exposed to 0.5, 1 and 2.5 mg/mL of BJe up to 96 h or with 10 nM of PMA. We therefore examined the cells and investigated their morphology with an inverted phase-contrast microscope Zeiss Primo Vert (Zeiss, Milan, Italy) at 400× magnification, supplied with AxioCam ERc5s camera, by which photos were captured.

### Cell surface antigen detection

Flow cytometry techniques were employed to detect cell surface markers of differentiation^27^. Briefly, THP-1 cells (3 × 10^5^ cells/mL), were seeded in 6-well plates. The day after, they were either exposed to 0.5, 1 and 2.5 mg/mL of BJe or 10 nM of PMA up to 96 h (by renewing treatment after 48 h). After the incubation period, cells were collected, centrifuged, and washed with cold PBS. Cell pellets were resuspended in 100 µL of PBS with anti-clusters of differentiation (CD)68 antibody conjugated with allophycocyanin (APC) (Miltenyi Biotec, Bergisch Gladbach, Germany), anti-CD11b antibody APC-conjugated (Miltenyi Biotec) or anti-CD14 antibody fluorescein-5-isothiocyanate (FITC)-conjugated (Beckman Coulter, Milan, Italy) and incubated at room temperature for 30 min in darkness. Thereafter, samples were analyzed by a Novocyte 2000 cytofluorimeter (ACEA Biosciences Inc., San Diego, CA, USA), with a minimum of 10,000 events being recorded.

### Western blotting analysis

In order to evaluate protein expression, THP-1 cells were plated onto 100 mm Petri dishes at a density of 1 × 10^6^ cells/dish (5 × 10^5^ cells/mL) and, the day after, exposed to 0.5, 1, 2.5 mg/mL of BJe for 48 h. After treatment, cells were harvested, washed with cold PBS and lysed by adding RIPA buffer (Sigma-Aldrich) containing 1% of protease and phosphatase inhibitor cocktail (Sigma-Aldrich). The lysed cells were centrifuged (12,000 g at 4 °C for 15 min) and the supernatant was collected and placed in a fresh tube kept on ice. Protein concentration was determined employing a Bio-Rad Protein Assay (Bio-Rad Laboratories, Milan, Italy) as described before^28^ by using bovine serum albumin as standard. Subsequently, 30 µg/well of proteins were separated by 10% or 12% sodium dodecyl sulphate–polyacrylamide gel electrophoresis (SDS-PAGE), and then electro-transferred onto polyvinylidene difluoride (PVDF; Merck Millipore, Darmstadt, Germany) membranes. After blocking with PBS containing 5% (*w*/*v*) non-fat dry milk for 1 h at room temperature, membranes were incubated at 4 °C overnight with the following primary antibodies: anti-extracellular signal regulated kinase 1/2 (ERK1/2), anti-phospho-ERK1/2, anti-c-Jun N-terminal kinase (JNK), anti-phospho-JNK, anti-p38, anti-phospho-p38, anti-microtubule-associated protein light chain 3 (LC3) A/B and anti-beclin-1 (Cell Signaling Technology, Danvers, USA), diluted 1:1000 in milk or BSA, and mouse monoclonal anti-β-actin-horseradish peroxidase (HRP) conjugated (Sigma- Aldrich), diluted 1:50,000.

Thereafter, membranes were washed three times with Tris-buffered saline containing 0.1% of Tween® 20 detergent (TBS-T) and probed for 2 h at room temperature with HRP-conjugated anti-goat or -rabbit IgG secondary antibodies (1:5,000; Sigma-Aldrich).

Chemiluminescence of protein bands was acquired employing ECL Prime Western Blotting Detection Reagent (GE Healthcare, Fischer Scientific, Milan, Italy) for 5 min and captured by a chemiluminescent detection system C-Digit Blot Scanner (Li-COR Bioscience, Lincoln, NE, USA). Image Studio software (Li-COR Bioscience) was employed to quantify protein bands. For each sample, β-actin was used as housekeeping protein to normalize signals intensity.

### Cell proliferation assays

In order to determine cell proliferation in presence of BJe, we evaluated the 5-bromo-2′-deoxyuridine (BrdU) incorporation employing the BrdU Cell Proliferation Assay Kit (Merck Millipore), as described^29^. In brief, THP-1 cells were seeded in 96-well plates (3 × 10^5^ cells/mL) and, after 24 h, they were incubated with fresh culture medium (negative control cells) or with medium supplemented with increasing dilution of BJe, ranging from 0 to 2.5 mg/mL. After 24, 48, 72 and 96 h, cells were exposed to BrdU for 2 h, then fixed and washed, prior to add the anti-BrdU HRP-conjugated antibody. At the end, the provided substrate was added, and the reaction stopped in order to detect results with an iMark™ microplate reader at 450 nm (Bio-Rad Laboratories). Results were reported as percentage of cell proliferation compared to untreated cells. The assay was repeated three times and performed using octuplicates.

For cell count assay, THP-1 cells (3 × 10^5^ cells/mL) were treated as for the BrdU incorporation assay in 6-well plates. At the end of the treatments, aliquots of cells were put in a Neubauer hemocytometer chamber, and cells were manually counted with a Zeiss Primo Star optical microscope^30^. Results were reported as number of cells and assays repeated three times and performed in triplicates.

### Statistical analyses

The one-way or two-way analyses of variance (ANOVA) were employed, based on the assay. Results are reported as mean ± standard error of the mean (SEM). Multiple comparisons of the means of the groups were carried out by the Dunnett’s multiple comparison test (GraphPAD Prism Software). P values less than or equal to 0.05 were regarded as statistically significant.

## Results

### BJe induced cell differentiation in THP-1 cells

#### Effect of BJe on NBT-reducing activity

When myeloid cells undergo maturation, a respiratory burst occurs after they are challenged with PMA, hence being able to reduce the NBT salt into blue insoluble diformazan deposits^31^. This phenomenon is regarded as an initial sign of differentiation. However, it has been observed that a higher number of immature cells could partly reduce NBT salt. This can conceal the high absorption produced by a fewer number of differentiated cells, since they stopped proliferating as part of their maturation process. For this reason, we coupled a viability cell assay, like the MTT test, with the NBT one, thus calculating the NBT/MTT ratio to measure cell differentiation rate in THP-1 cells treated with BJe, normalized to their viability.

**Fig. 1:**
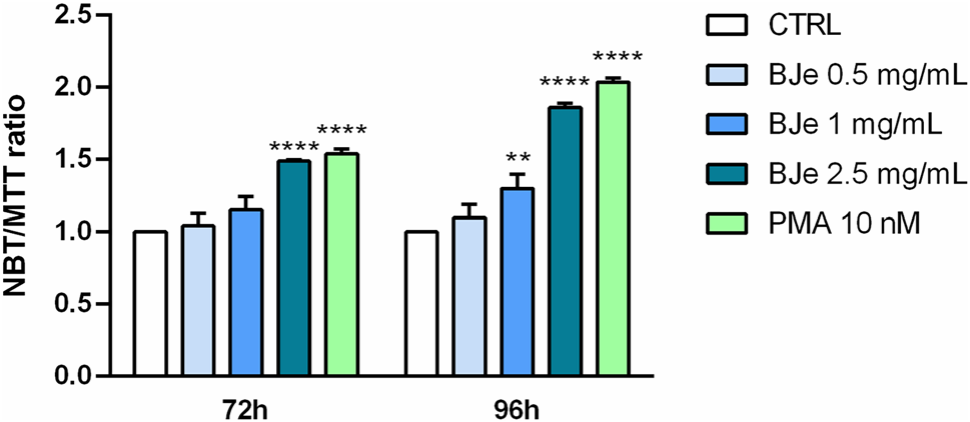
NBT/MTT ratio in THP-1 cells after 72 h and 96 h of exposure to BJe. Cells were treated with BJe (0.5, 1 and 2.5 mg/mL) for 72 and 96 h. Cell differentiation was assessed by NBT test, whereas viability by MTT test. Results are expressed as mean ± SEM of the ratio between NBT and MTT reduction, and compared to untreated cells (control, CTRL). Three different experiments were carried out by testing each concentration eight-fold (N = 24). **p < 0.01, ***p < 0.001 and ****p < 0.0001 versus CTRL.

The exposure to BJe for 24 and 48 h did not increase NBT/MTT ratio in THP-1 cells (data not shown). Conversely, after 72 and 96 h, BJe treatment augmented the NBT/MTT ratio, as displayed in Fig. 1, yet only the concentration 2.5 mg/mL produced significant results already at 72 h (1.49 ± 0.009-fold-increase, *p* < 0.0001 vs. CTRL) with a more marked effect after 96 h of treatment (1.86 ± 0.03-fold-increase, *p* < 0.0001 vs. CTRL). To note, most of the cells that survived after treatment with 2.5 mg/mL of BJe, for the indicated time points, were mature (i.e., differentiated). Nevertheless, also the treatment with BJe 1 mg/mL brough a significant increase of the NBT/MTT ratio only after 96 h (1.29 ± 0.099-fold-increase, *p* < 0.01 vs. CTRL). On the contrary, treatment with 0.5 mg/mL BJe did not exert any significant effect. As expected, exposure to 10 nM of PMA, used as positive control, significantly increased NBT/MTT ratio (1.54 ± 0.03-fold increase, *p* < 0.0001 vs. CTRL after 72 h, and 2.03 ± 0.03-fold increase, *p* < 0.0001 vs. CTRL after 96 h).

#### BJe induced morphological changes and adherence in THP-1 cells

**Fig. 2:**
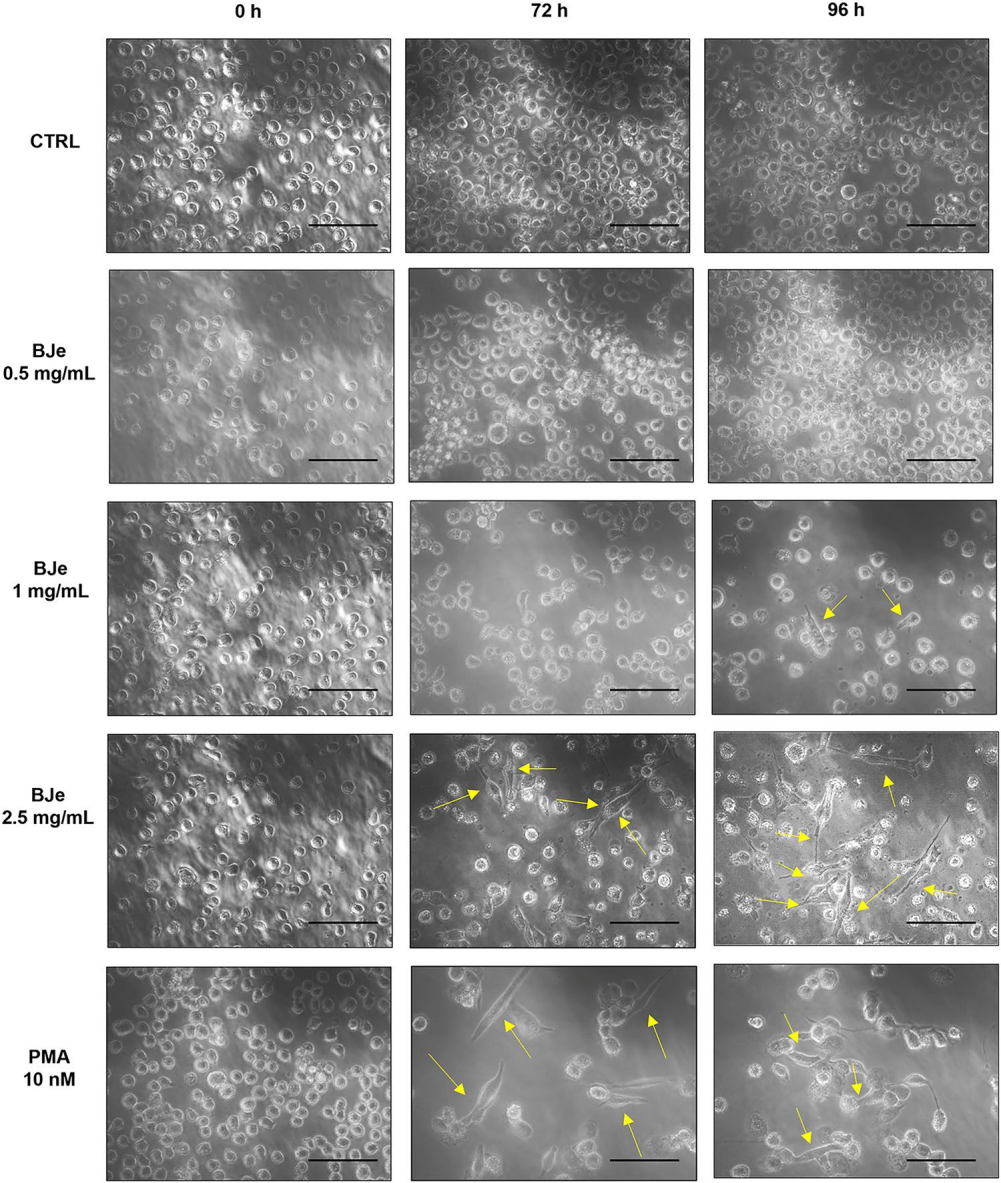
Morphological changes induced by BJe in THP-1 cells. Cells were exposed to 0.5, 1 and 2.5 mg/mL of BJe up to 96 h or with 10 nM of PMA. In comparison with unexposed monocytes (CTRL), differentiated macrophage-like cells tend to adhere to the surface of the cultivation dishes, as indicated by yellow arrows. Cells were examined by light microscopy (400× magnification; scale bar 100 µm). Pictures are representative of three different experiments.

The effects of the treatment with BJe 0.5, 1 and 2.5 mg/mL in THP-1 cells for 24, 48, 72 and 96 h were examined by light microscopy to observe cell morphology. Images for the longer timings of treatment are shown in Fig. 2. PMA 10 nM was used as positive control.

During shorter treatment times (24–48 h), both BJe-treated and untreated cells presented similar cell morphology (smooth membrane surface as well as round shape; data not shown). Nevertheless, after 72 and 96 h of incubation, 2.5 mg/mL of BJe and 10 nM of PMA caused a clear morphologic change of THP-1 cells into a macrophage-like one, presenting different cell shapes and capability to adhere to the surface of dishes, compared to untreated cells (Fig. 2). Interestingly, the 1 mg/mL brought an early sign of differentiation at 96 h, as some cells started to change their morphology; however, the 0.5 mg/mL did not elicit any appreciable change.

#### BJe augmented levels of cell surface differentiation markers

**Fig. 3:**
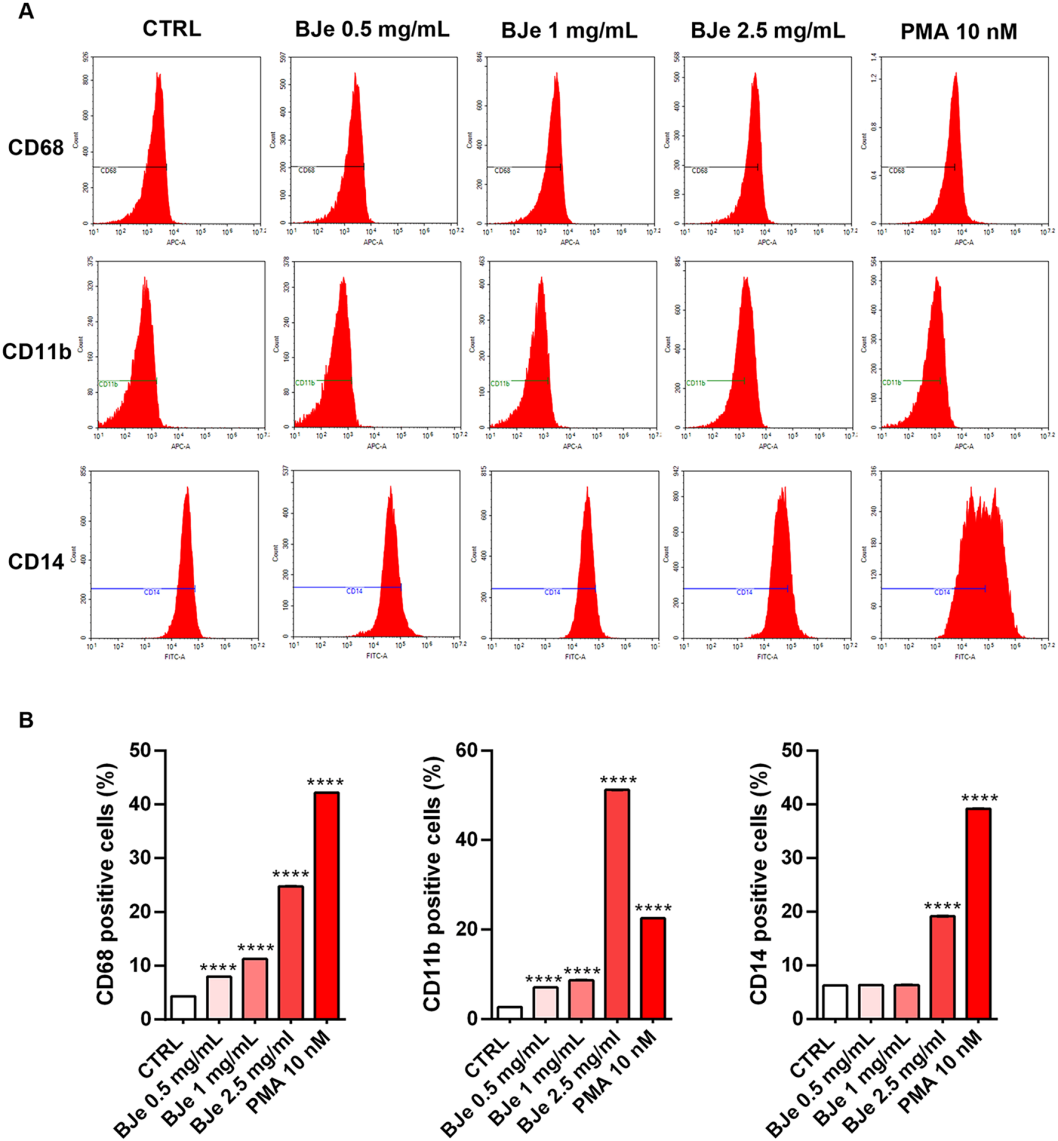
BJe induced THP-1 cell differentiation as indicated by the increase of cell surface markers of differentiation expression. (**A**) THP-1 cells were exposed to 0.5, 1 and 2.5 mg/mL of BJe or PMA 10 nM for 96 h and levels of CD68, CD11b and CD14 were determined by flow cytometry. (**B**) Percentages of CD68^+^, CD11b^+^ ad CD14^+^ cells are reported as the mean ± SEM of three different experiments (N = 3). ****p < 0.0001 verusus control (CTRL).

With the aim of further confirming THP-1 cell differentiation induced by BJe, we detected cell surface differentiation antigens by flow cytometric techniques. Therefore, we assessed the expression of monocyte-to-macrophage differentiation marker CD68, myeloid differentiation marker CD11b and monocytic maturation marker CD14^32^. As shown in Fig. 3, after 96 h of treatment, the 0.5, 1 and 2.5 mg/mL of BJe significantly enhanced the expression of CD68 (7.99 ± 0.0002%, 11.29 ± 0.0002% and 24.79 ± 0.0003%, *p* < 0.0001 vs. CTRL, respectively) and CD11b (7.09 ± 0.05%, 8.69 ± 0.03%and 51.21 ± 0.02%, *p* < 0.0001 vs. CTRL, respectively). Treatment with 2.5 mg/mL of BJe for 96 h significantly augmented CD14 levels (19.18 ± 0.05%, *p* < 0.0001 vs. CTRL), conversely exposure to 1 mg/mL of BJe did not yield any significant effect. As expected, 10 nM of PMA significantly increased CD68, CD11b and CD14 surface markers expression (42.18 ± 0.002%, *p* < 0.0001 vs. CTRL; 22.53 ± 0.02%, *p* < 0.0001 vs. CTRL; 39.19 ± 0.05%, *p* < 0.0001 vs. CTRL, respectively).

### BJe triggered autophagy in THP-1 cells

Since the induction of autophagy is critical for monocyte to macrophage differentiation^33^, as well as representing a key factor for an effective differentiation-inducing protocol in myeloid leukemia cells^34^, we evaluated whether BJe was capable of modulating proteins related to autophagy in THP-1 cells.

**Fig. 4:**
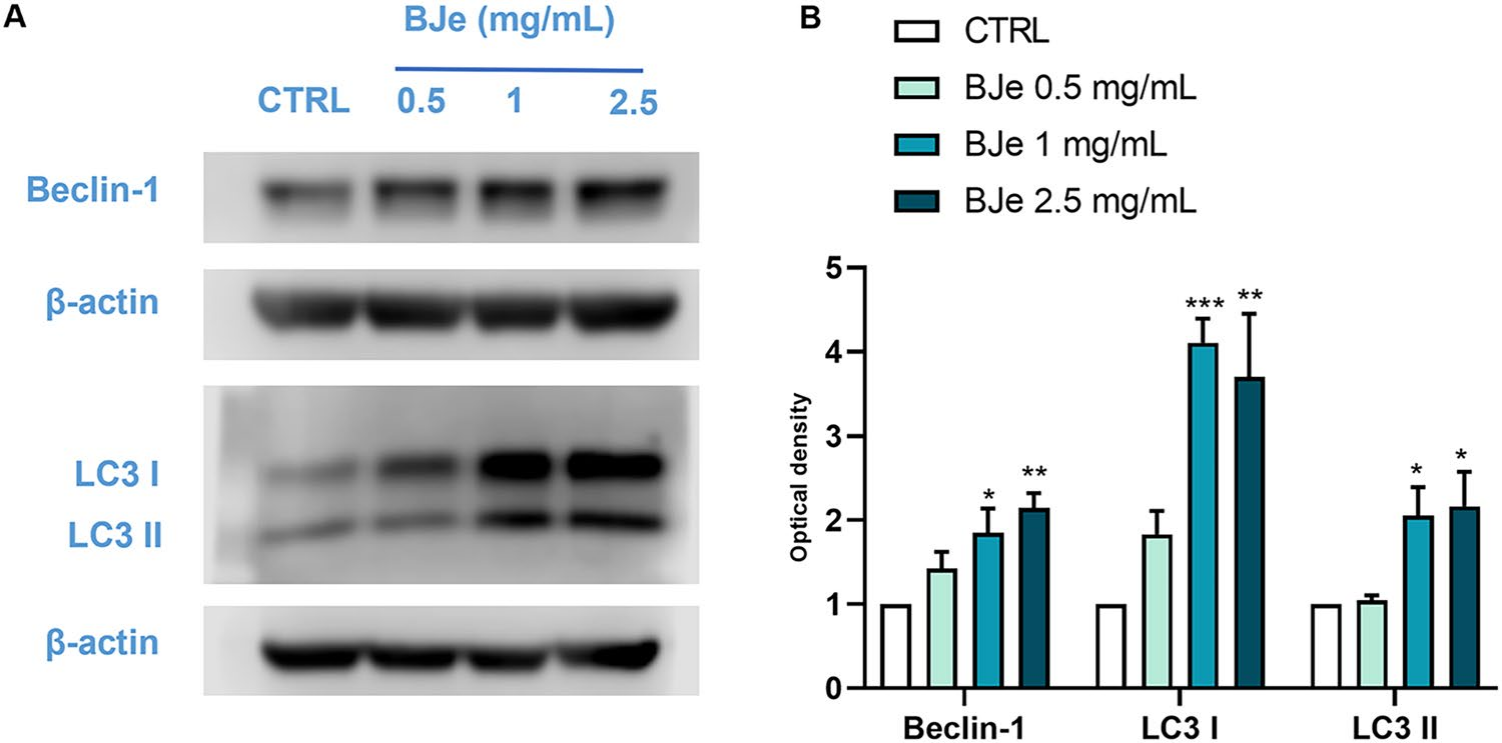
Modulation of autophagy-related protein levels after BJe treatment in THP-1 cells. Cells were treated with BJe ranging from 0.5 to 2.5 mg/mL for 48 h before being processed for protein expression studies through Western blotting. (**A**) Representative immunoblots of three different experiments are shown as well as their densitometric analyses, on the right (**B**). Protein expression of Beclin-1, LC3 I and LC3 II was normalized against β-actin. Results are extrapolated as fold change with respect to unexposed cells and reported as mean ± SEM of values obtained in at least three independent experiments (N = 3 for Beclin-1 and N = 4 for LC3 I–II). *p < 0.05, **p < 0.01 and ***p < 0.001 versus control (CTRL).

Protein levels of Beclin-1, also known as autophagy-related 6 homolog (ATG6), that is involved in the initial stage of the autophagic process, were significantly increased after exposure to 1 and 2.5 mg/mL of BJe for 48 h (Fig. 4) by 1.85 ± 0.28-fold and 2.14 ± 0.18-fold (*p* < 0.05 and *p* < 0.01 vs. CTRL, respectively). Conversely, treatment with the 0.5 mg/mL concentration did not induce any significant change in Beclin-1 protein levels.

LC3 is one of the principal components of the autophagosome; its cytosolic soluble form (LC3-I) is processed, lipidated, as well as recruited to the membrane of the autophagosome (LC3-II) during the final stages of autophagy. Western blot analyses, employing an antibody that recognizes both protein forms, showed a clear increase in expression levels of both LC3 I and II after treatment with BJe (Fig. 4). In particular, treatment with 1 and 2.5 mg/mL concentration significantly augmented LC3-I levels by 4.11 ± 0.29-fold (*p* < 0.001, vs. CTRL) and 3.70 ± 0.75-fold (*p* < 0.01, vs. CTRL), respectively. Levels of LC3-II were increased of 2.05 ± 0.34-fold (*p* < 0.05, vs. CTRL) and 2.16 ± 0.41-fold (*p* < 0.05, vs. CTRL) after incubation for 48 h with 1 and 2.5 mg/L, respectively. The 0.5 mg/mL did not elicit any significant result.

### BJe induced mitogen-activated protein kinases (MAPKs) phosphorylation in THP-1 cells

Recent findings pointed out that mitogen-activated protein kinases (MAPKs) can modulate autophagy and, consequently, induce differentiation in leukemic cells^35^. Thus, we investigated the effect of BJe on protein expression of phospho-ERK, phospho-JNK and phospho-p38.

**Fig. 5:**
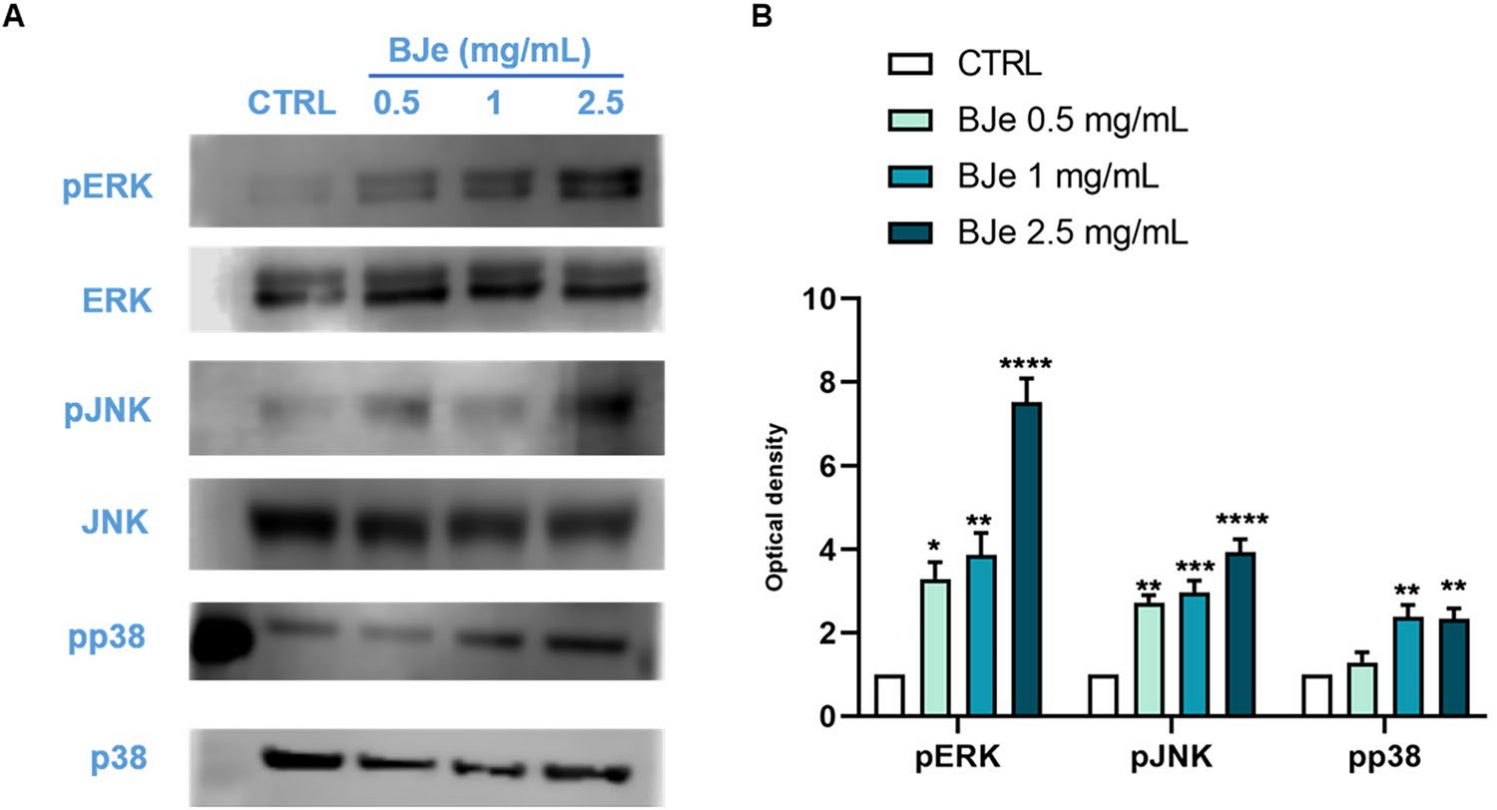
Effect of BJe on MAPKs protein expression in THP-1 cells. Cells were exposed to BJe at 0.5, 1 and 2.5 mg/mL for 48 h, respectively and were then processed for protein level assessment determined by Western blotting. On the left (**A**), immunoblots representative of three independent experiments are shown, while their densitometric analysis is displayed on the right (**B**). Levels of phosphorylated JNK, ERK and p38 were expressed as a ratio of phosphorylated forms over their total protein concentration. Results were extrapolated as fold change versus untreated cells, which are assigned as 1 arbitrarily and expressed as mean ± SEM, of three different experiments (N = 3). *p < 0.05, **p < 0.01, ***p < 0.001 and ****p < 0.0001 versus control (CTRL).

As shown in Fig. 5, exposure to 0.5, 1 and 2.5 mg/mL of BJe for 48 h increased the levels of phospho-ERK of 3.30 ± 0.4-fold, 3.87 ± 0.52-fold and 7.52 ± 0.57-fold (*p* < 0.05, *p* < 0.01 and *p* < 0.0001, vs. CTRL respectively) with respect to the unexposed cells. Treatment with 0.5 mg/mL of BJe significantly enhanced the levels of phospho-JNK of 2.73 ± 0.18-fold, (*p* < 0.01 vs. CTRL), whereas 1 mg/mL of 2.98 ± 0.27-fold (*p* < 0.001, vs. CTRL) and 2.5 mg/mL of 3.94 ± 0.31-fold (*p* < 0.0001, vs. CTRL). In addition, we observed a significant increase in phospho-p38 levels after treatment with both 1 and 2.5 mg/ml of BJe by 2.39 ± 0.28-fold (*p* < 0.01 vs. CTRL) and 2.34 ± 0.25-fold (*p* < 0.01 vs. CTRL), respectively (Fig. 5).

### BJe decreased proliferation of THP-1 cells

**Fig. 6:**
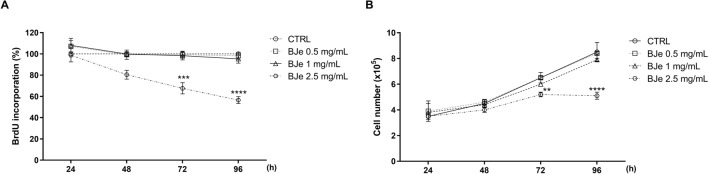
Effect on cell proliferation of BJe in THP-1 cells. Cells were exposed to 0.5, 1 and 2.5 mg/mL of BJe for 24, 48, 72 and 96 h. Cell proliferation was determined by incorporation of BrdU (**A**) and cell count (**B**) tests. Data are reported as percentages ± SEM of absorbance respect to values assessed in the untreated cultures (control, CTRL), while for cell count as number of cells (× 10^5^). Three experiments were carried out independently by testing each concentration eight-fold (N = 24) for BrdU incorporation, while three experiments testing three replicates (N = 9) for cell count. **p < 0.01, ***p < 0.001 and ****p < 0.0001 versus control (CTRL).

Given the possible role of differentiating agents to reduce the proliferation of leukemic cells, we evaluated whether the differentiation activity exerted by BJe was accompanied by an anti-proliferative effect. Therefore, we investigated the effects on cell proliferation exerted by BJe through the incorporation of BrdU test. As displayed in Fig. 6A, the highest concentration of BJe (2.5 mg/mL) significantly decreased proliferation only at 72 (− 35.4 ± 5.2%, *p* < 0.001 vs. CTRL) and 96 h (− 55.5 ± 3.3%, *p* < 0.0001 vs. CTRL). After any of the timing points tested neither 0.5 nor 1 mg/mL of BJe altered cell proliferation during the observed period.

These results were also confirmed by cell count (Fig. 6B), where BJe 2.5 mg/mL significantly reduced the number of cells at both 72 h (− 20 ± 2.3%, *p* < 0.01 vs. CTRL) and 96 h (− 40 ± 3.3%, *p* < 0.0001 vs. CTRL). Even for cell count, the 0.5 and 1 mg/mL did not decrease cell number at any of the tested timing points.

## Discussion

The connection between nutrition and human health has always stimulated the interest of the scientific community. This is due to the ever-increasing evidence supporting the protective effects of natural compounds, commonly retrieved from the diet, in the management of different diseases, including neoplasms. In particular, *Citrus* fruits, which are some of the principal constituents of the Mediterranean diet, represent a valuable source of precious bioactive molecules endowed with a wide plethora of beneficial properties^36^. Several studies highlighted their potentiality as anti-tumor agents, thus suggesting that *Citrus* fruits and their flavonoids can hamper the development of cancer^37^. *Citrus* fruits flavonoids have been shown to counteract different processes of tumorigenesis^38^ that can underlie their antileukemic effects witnessed both in vitro^39^ and in vivo^40^. Amongst *Citrus* fruits, bergamot has been lately widely studied for its anti-cancer properties. In particular, we documented that the antiproliferative effect of BJ was due to its flavonoid fraction, since BJe was able to hamper proliferation and trigger apoptosis in human colorectal carcinoma HT-29 cells by multiple mechanisms^24^, as well as the spontaneous formation of tumors in Pirc rats^22^. Moreover, we also showed the safety of BJe since it did not hamper the cell viability of human peripheral blood mononuclear cells (PBMCs)^23^.

In AML, the normal differentiation process is damaged, and the cells proliferate constantly without entering terminal apoptosis^41^. The idea of the differentiation therapy lies on eliminating differentiation arrest, hence forcing cells to lessen their proliferation^42^. Even more, beneficial effects of treatment could be more easily monitored, as hematopoietic cells show distinct morphological features during their differentiation process, as well as expressing diverse surface markers during differentiation^43^. The success of differentiation therapy pointed out by ATRA and/or ATO use is valid for one particular subtype of leukemia^44^, the acute promyelocytic leukemia (APL), defined by the French–American–British (FAB) classification as M3 subtype of AML, accounting for 5–10% of total AML cases. In addition, both ATRA and ATO can often cause the so-called “differentiation syndrome”, hence diminishing the overall patient’s compliance. Therefore, the search for new and safer differentiation inducers is absolutely required, principally for leukemia types other than APL, including those belonging to M4 and M5 subtypes, commonly associated with unfavorable prognosis^45^.

In the last decades, several findings highlighted the pro-differentiating properties of flavonoids in different leukemia cells^16–18^, including those from *Citrus*^46^. Moreover, it has been shown that the inhibition of histone deacetylases, such as SIRT2, overexpressed in AML^47^, could be responsible of an antileukemic effect following the differentiation induction^48^. Of note, very recently, we demonstrated that BJe, as well as its main flavonoids, were able to inhibit both SIRT2 enzymatic activity and its expression in THP-1 cells^23,49^.

On these bases, we investigated whether BJe was able to induce differentiation in THP-1 cells, a cell line belonging to the M5 subtype and presenting the mixed lineage leukemia (MLL) gene rearrangements (MLLr), generally associated with poor prognosis^45^.

A hallmark of differentiation is the ability of differentiating cells to undergo oxidative burst in presence of an exogenous “stimulus”. In physiological conditions, this happens when granulocytes or monocytes meet harmful microorganisms thus producing reactive oxygen species (ROS) in order to defeat them^31^. The presence of pathogens can be simulated in vitro by using an external stimulus, such as N-formyl-methionyl-Ieucyl-phenylalanine, bacterial lipopolysaccharide (LPS) or, more commonly PMA. Consequently, the exposure to pathogens activates the oxidative burst enzymes in mature cells, leading to the release of superoxide anions that can reduce the soluble yellow NBT salt into insoluble formazan blue deposits^50^. Therefore, we investigated whether BJe was capable of inducing cell differentiation, assessing the reduction of NBT salt after stimulation with PMA. However, since also immature cells can partly reduce NBT, despite to a lesser extent than mature ones, we coupled an MTT test, performed in parallel, to normalize our results with the relative number of vital cells. For the first time, we showed that BJe is able to induce differentiation in THP-1 cells, as demonstrated by the increase of the NBT/MTT ratio, suggesting that the highest concentration of BJe behaved as PMA, the gold standard of the differentiating agents. Our findings are in accordance with what was observed by Kawaii et al., who reported that the extracts of diverse *Citrus* juices (i.e., sweet lime, Shiikuwasha and bergamot) have been shown to exert an NBT reducing effect in HL-60 APL cells^51^.

Another important characteristic of hematologic differentiating cells is that they express definite surface markers, called CDs, that resemble a specific hematopoietic differentiation stage. Interestingly, in our study, BJe was capable of increasing the expression of monocyte to- macrophage differentiation marker CD68, myeloid differentiation marker CD11b and monocytic maturation marker CD14 in THP-1 cells, hence indicating macrophage differentiation^32^, and reinforcing results obtained from the NBT test. The differentiation capability of BJe is further sustained by morphological observations, in which differentiated cells, attached to the surface, showed macrophage-like morphology with flat shape and the presence of long pseudopodia extending from the soma of the cells. Notably, the highest concentration of BJe displayed a greater differentiating capability than PMA as regards cell morphology.

Autophagy is a catabolic process that is involved in the lysosomal degradation of cytoplasmic material, relevant for the preservation of cellular homeostasis^52^. Recently, it has been considered a double-edged sword in acute leukemias because it can either suppress or promote tumor survival and growth, depending on the cell functional status and context. In AML, the ambivalent role of autophagy is exploited in targeted therapies or chemotherapy resistance, depending on the drug employed^53^. However, several studies considered it a key factor for an effective differentiation-inducing regimen in leukemia cells^34^. Indeed, numerous researchers verified that one of the mechanisms responsible of ATO- and/or ATRA-induced differentiation of APL cells was activation of the autophagic process^54^. In particular, Isakson and collaborators observed that ATRA-induced autophagy provokes granulocytes differentiation in APL cells, through a mechanism involving the demolition of the oncogenic fusion protein PML-RARα, amongst others^55^. Regarding non-APL cells (i.e., THP-1 ones), Benjamin and coworkers^34^ showed that the combination of ATRA and valproic acid induced autophagy and differentiation in both APL and non-APL cells. The involvement of autophagy in their combination treatment was further confirmed since shRNA knockdown of the autophagy regulators TFEB and ATG7 lessened both autophagy and differentiation, hence highlighting its importance in both processes. Interestingly, we witnessed a significant increase of protein levels of both LC3 I and II, pivotal protein of the autophagic machinery, as well as those of beclin-1, another autophagy-related marker involved in the early stage of the autophagic machinery, hence suggesting that the pro-differentiating activity of BJe proceeds through the unleashing of autophagy.. Notably, it was shown that the treatment of THP-1 cells with BJe diminished phosphorylation levels of AKT^23^, a kinase inhibitor of the autophagic process^56^, thus sustaining the activation of autophagy mediated by BJe.

Among the regulators of autophagy, MAPKs play a pivotal role and are known to modulate this machinery in different cancer models^57^. In leukemic cells, Yang and co-workers found that brassinin, a phytoalexin retrieved from cruciferous vegetables, induced autophagy by activating MAPK signaling pathway in K562 chronic myelogenous leukemia cells^58^, as Qin and colleagues who showed that hinokiflavone, a biflavonoid present in *Selaginella tamarisina*, induced autophagy in the same cells through MAPK/NF-κB signaling pathway^59^. Interestingly, MAPKs are also involved in the process of cellular differentiation, also in various types of leukemic cells^60,61^. For instance, Procházková and colleagues observed that co-treatment of HL-60 cells with tumor necrosis factor-alpha (TNF-alpha) and inhibitor of 5-lipoxygenase MK886 provoked cell differentiation through the activation of p38 and JNK pathways, while Ju and co-workers witnessed that exposure to *Nardostachys chinensis* extract stimulated ERK pathway and triggered the differentiation of HL-60 cells into granulocytes. Taken together, this evidence suggests the existence of a link between MAPKs activation and induction of autophagy, which in turn leads to leukemic cell differentiation. Indeed, Mandic and collaborators highlighted how MAPKs, autophagy, and differentiation can be mutually correlated in leukemic cells^35^. In particular, they suggested that MAPKs can modulate PMA-induced macrophage differentiation in HL-60 cells through the regulation of autophagy by FOXO1/3 and TFEB nuclear translocation, together with the dissociation of beclin-1/Bcl2 complex. For these reasons, we investigated the effect of BJe on protein levels of phosphorylated ERK, JNK and p38 in THP-1 cells that we found significantly augmented, thus reinforcing our hypothesis on the putative connection between MAPKs phosphorylation and BJe pro-autophagic and pro-differentiating properties. Noteworthily, the different effect of BJe on MAPKs, we observed in the human colon cancer HT-29 cells^24^ may be attributable to the different function of MAPKs depending on the cell type^62^. For instance, the activation of ERK/MAPK signaling pathway in colon cancer cells can cause an increase in their proliferation^63^, while it may provoke cell death in certain leukemia cells, as a result of a terminal differentiation process^16–18^.

Based on the quali-quantitative composition of BJe^23^, we can hypothesize that the main players of the abovementioned effects we observed may be naringin and neohesperidin, being the wide majority of the whole extract. Indeed, it has been already demonstrated that, despite in different experimental models, naringin induced autophagy via activation of MAPK pathways in AGS cancer cells^64^, while neohesperidin was able to prompt in SJSA and HOS osteosarcoma cells towards autophagy via the activation of the JNK pathway^65^. Regarding their pro-differentiating activity, Takashi and co-workers claimed that naringin was not able to induce a significant differentiation effect, in terms of NBT reduction, in HL-60 cells^66^, whereas for neohesperidin there is no evidence. With this study, we are the first to describe the pro-differentiating effect of BJe and, indirectly, of its constituents together. This is in line with the fact that phytocomplexes are known to benefit considerably from the synergy between their constituents, as we have already demonstrated^8,49,67^. Consequently, the multitarget capability of phytocomplexes may have a larger impact than that of single molecules. The synergism in natural products is an excellent tool from a prospective therapeutic strategy since lower doses might yield bigger benefits^68^.

## Conclusions

In summary, we showed that BJe induced pro-differentiating and anti-proliferative effects in human leukemia monocytic THP-1 cells, unleashing autophagy and suggesting MAPKs as possible cross-talk in the differentiation process, thus pointing out its potential use in the area of differentiation therapy of AML.

### Supplementary Information

Supplementary Information.

## Data Availability

The data used to support the findings of this study are available from the corresponding author upon a suitable request.
