# Supplementary material for: The pro-differentiating capability of a flavonoid-rich extract of Citrus bergamia juice prompts autophagic death in THP-1 cells
Source: Sci Rep. 2024 Aug 28;14:19971. doi: 10.1038/s41598-024-70656-4 (PMC11358463; doi:10.1038/s41598-024-70656-4)
Supplement: Supplementary file 1 — Supplementary Information. [file 41598_2024_70656_MOESM1_ESM.pdf]

LC3 A/B

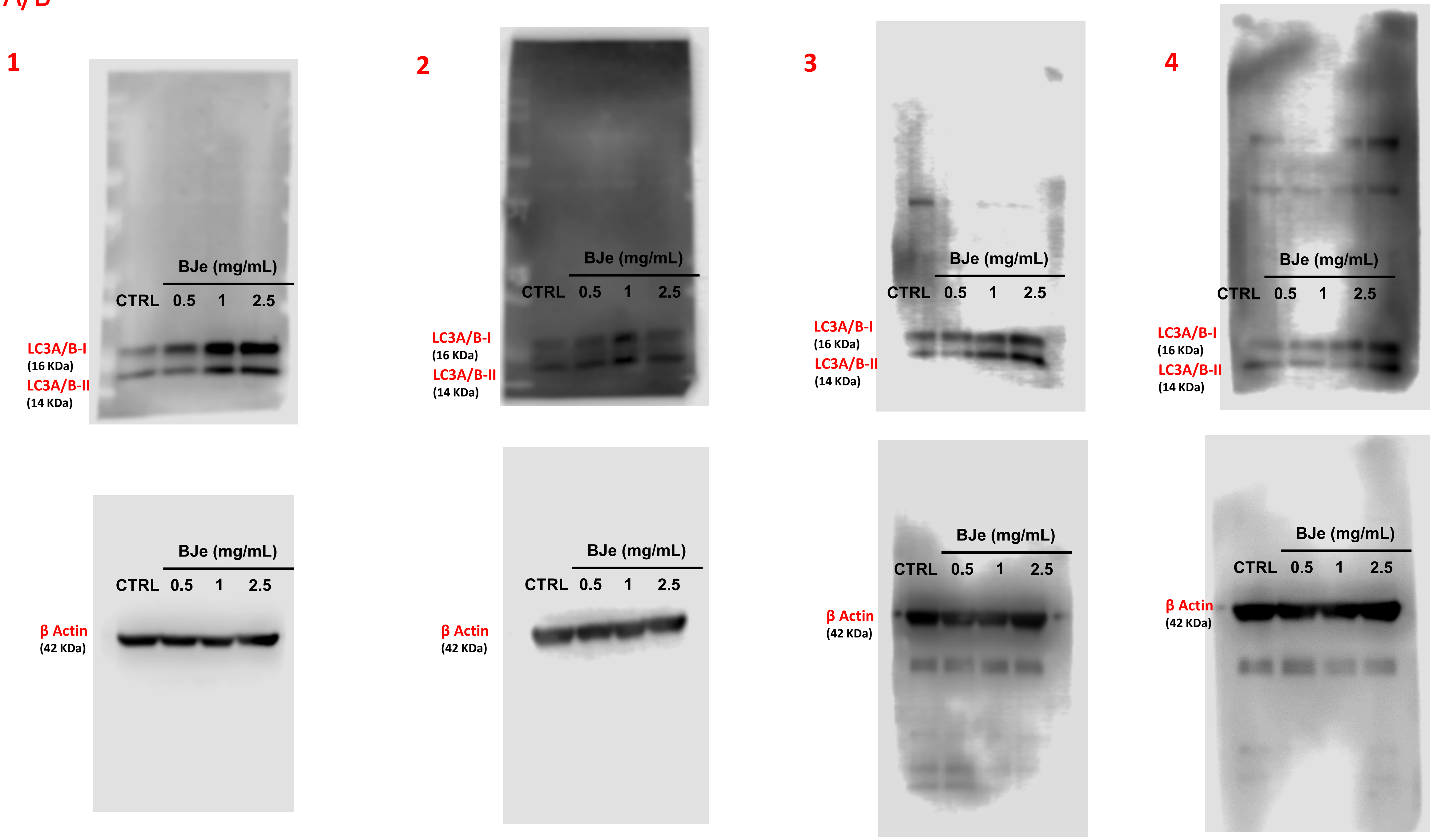
1
BJe (mg/mL)
CTRL 0.5 1 2.5
LC3A/B-I
(16 KDa)
LC3A/B-II
(14 KDa)
BJe (mg/mL)
CTRL 0.5 1 2.5
β Actin
(42 KDa)
2
BJe (mg/mL)
CTRL 0.5 1 2.5
LC3A/B-I
(16 KDa)
LC3A/B-II
(14 KDa)
BJe (mg/mL)
CTRL 0.5 1 2.5
β Actin
(42 KDa)
3
BJe (mg/mL)
CTRL 0.5 1 2.5
LC3A/B-I
(16 KDa)
LC3A/B-II
(14 KDa)
BJe (mg/mL)
CTRL 0.5 1 2.5
β Actin
(42 KDa)
4
BJe (mg/mL)
CTRL 0.5 1 2.5
LC3A/B-I
(16 KDa)
LC3A/B-II
(14 KDa)
BJe (mg/mL)
CTRL 0.5 1 2.5
β Actin
(42 KDa)

Beclin-1

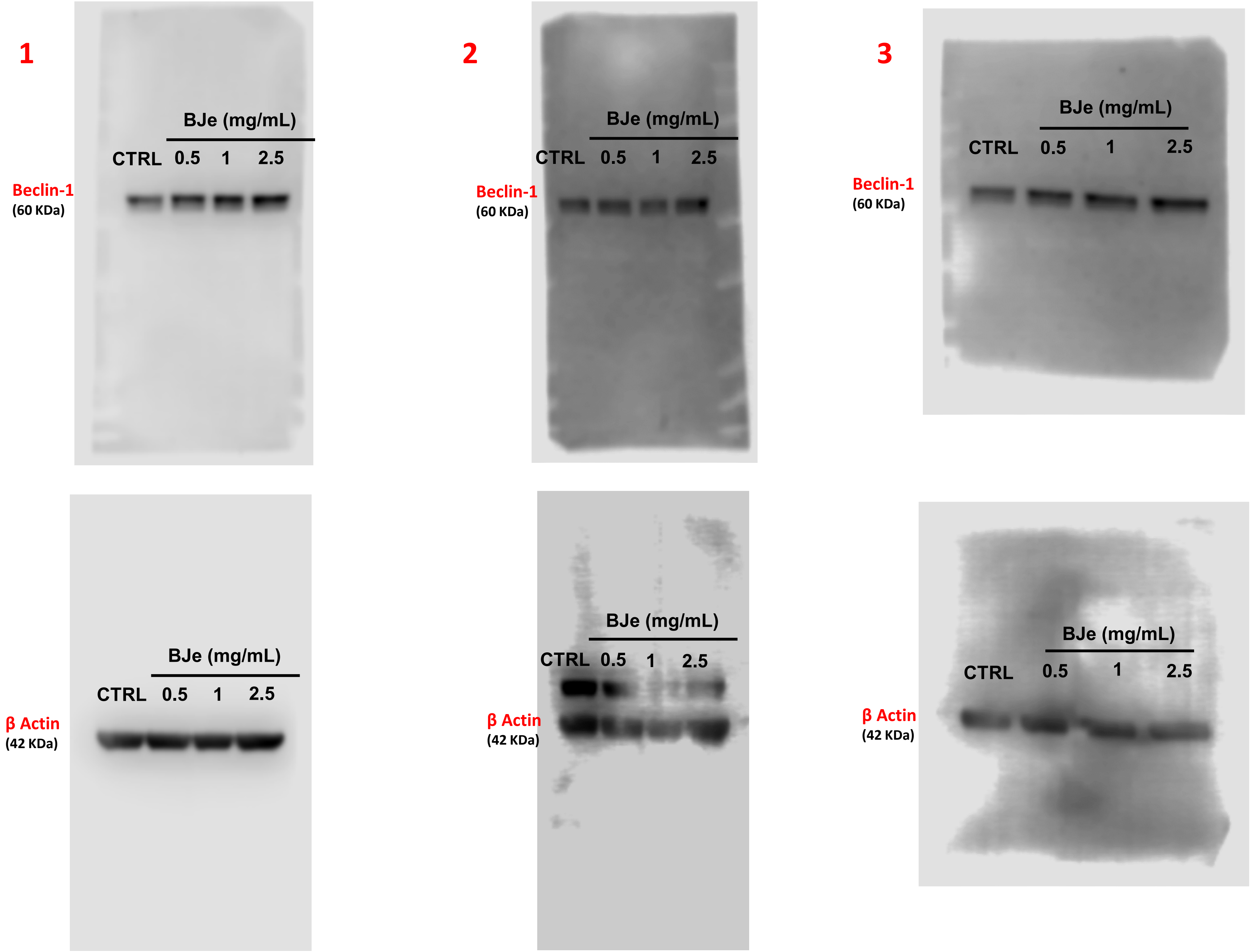

1
BJe (mg/mL)
CTRL 0.5 1 2.5
Beclin-1
(60 KDa)
BJe (mg/mL)
CTRL 0.5 1 2.5
β Actin
(42 KDa)
2
BJe (mg/mL)
CTRL 0.5 1 2.5
Beclin-1
(60 KDa)
BJe (mg/mL)
CTRL 0.5 1 2.5
β Actin
(42 KDa)
3
BJe (mg/mL)
CTRL 0.5 1 2.5
Beclin-1
(60 KDa)
BJe (mg/mL)
CTRL 0.5 1 2.5
β Actin
(42 KDa)

Phospho-ERK

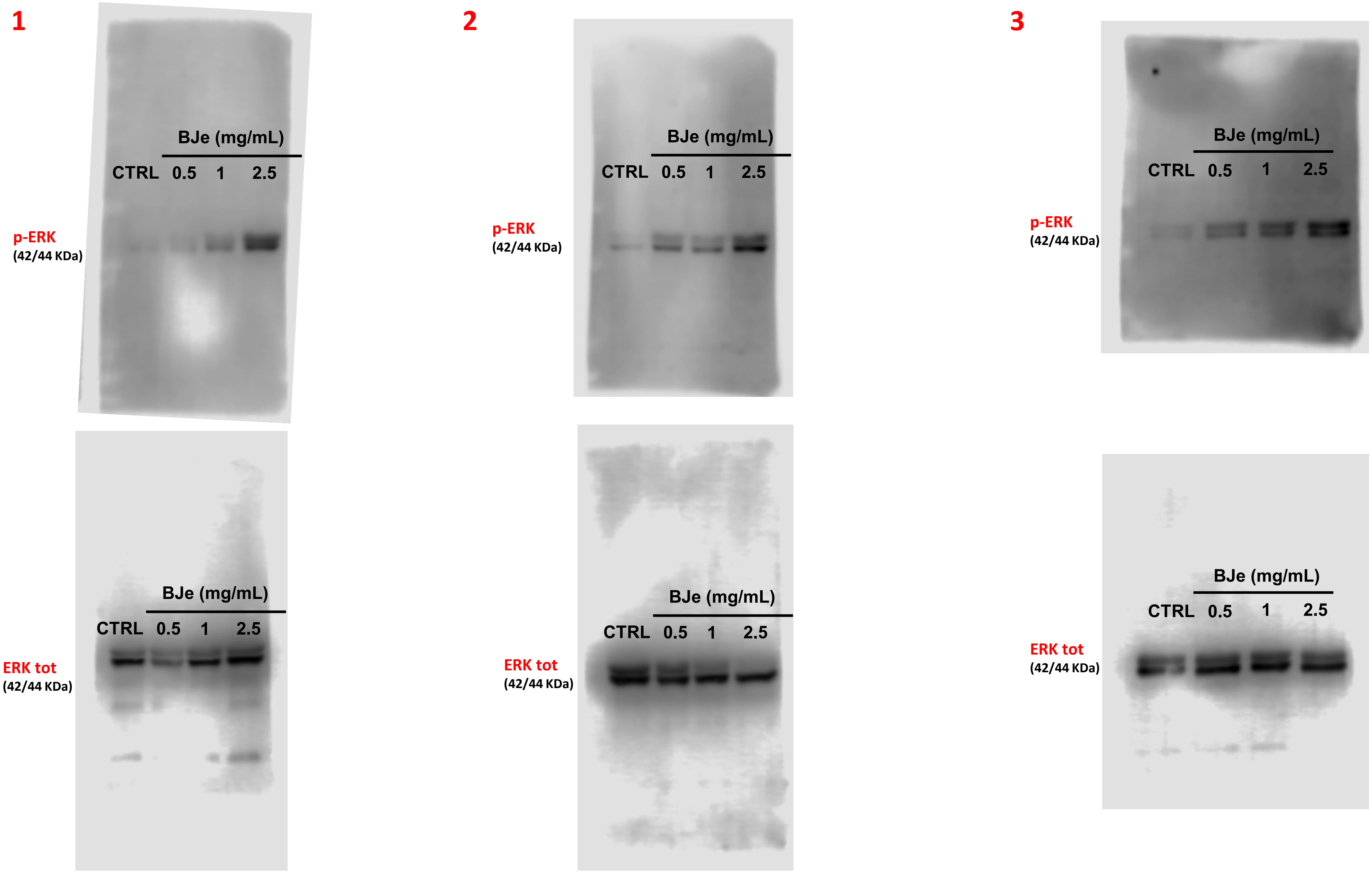

Phospho-JNK

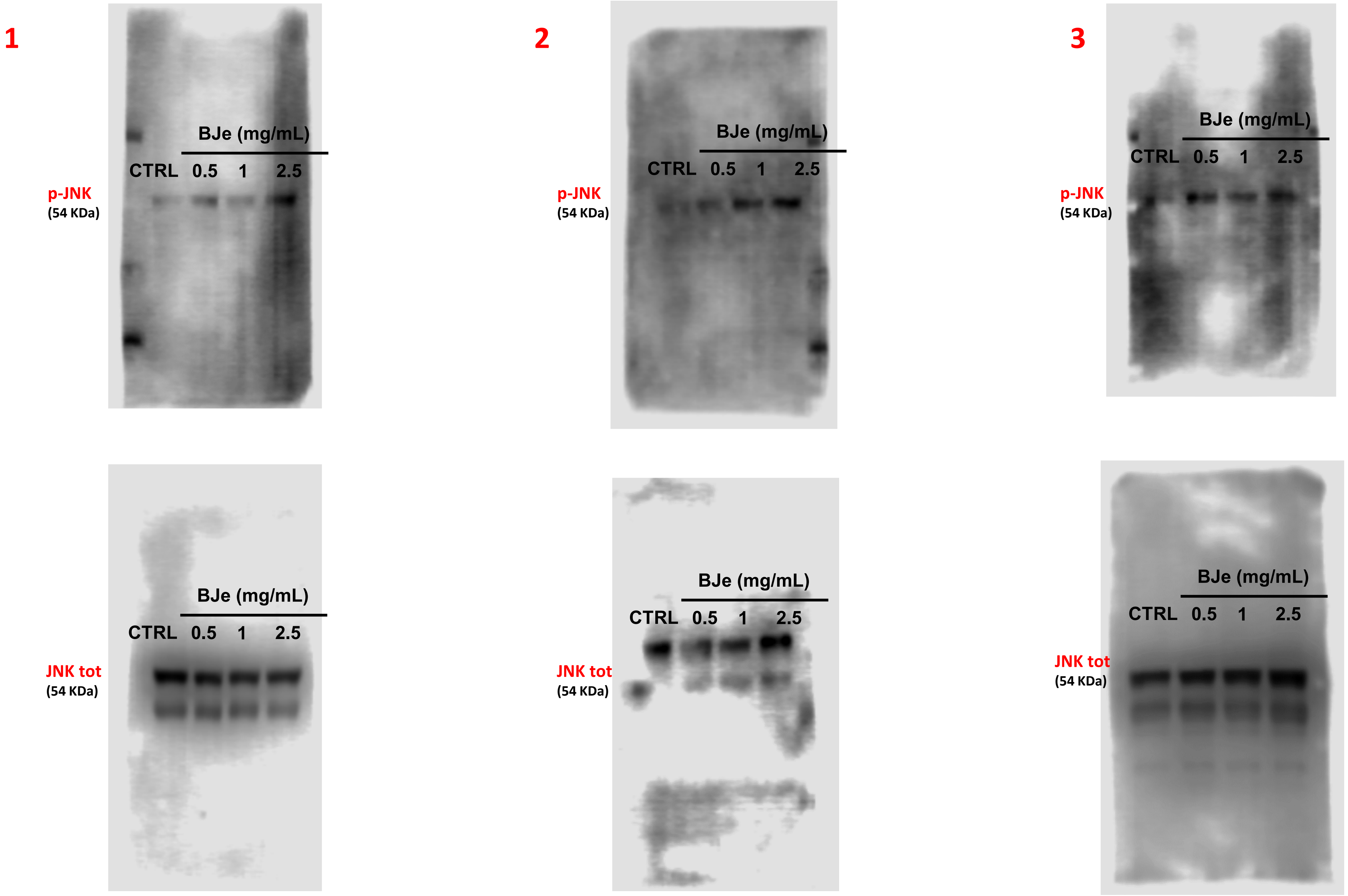

Phospho-p38

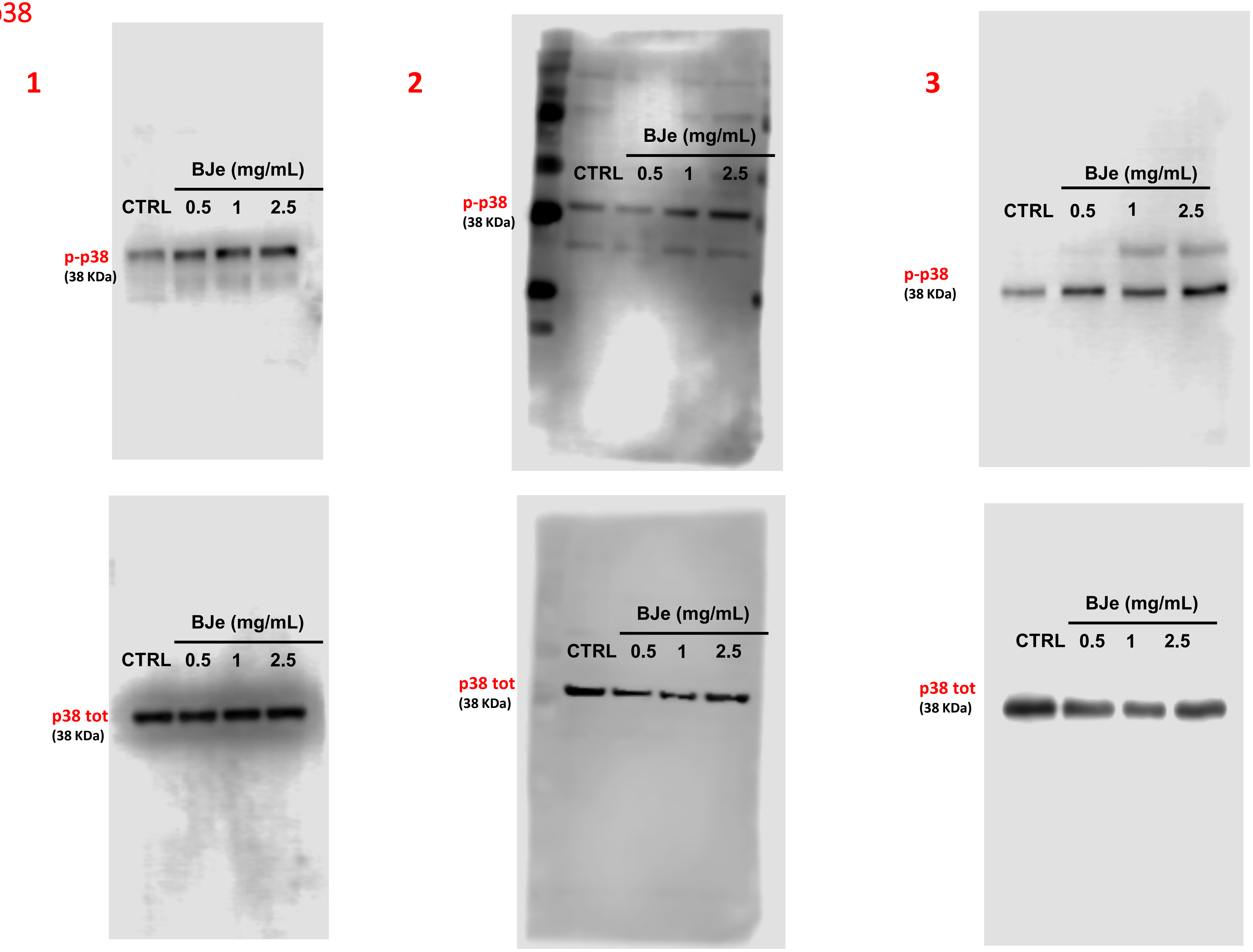
1
BJe (mg/mL)
CTRL 0.5 1 2.5
p-p38
(38 KDa)
BJe (mg/mL)
CTRL 0.5 1 2.5
p38 tot
(38 KDa)
2
BJe (mg/mL)
CTRL 0.5 1 2.5
p-p38
(38 KDa)
BJe (mg/mL)
CTRL 0.5 1 2.5
p38 tot
(38 KDa)
3
BJe (mg/mL)
CTRL 0.5 1 2.5
p-p38
(38 KDa)
BJe (mg/mL)
CTRL 0.5 1 2.5
p38 tot
(38 KDa)
